# ‘Handled with care’: Diffuse policing and the production of inequality in Amsterdam

**DOI:** 10.1177/1466138117696107

**Published:** 2017-02-28

**Authors:** Anouk de Koning

**Affiliations:** Radboud University, The Netherlands

**Keywords:** urban anthropology, security, policing, racialization, Europe, the Netherlands

## Abstract

The intersection of race and the criminal justice system has been a longstanding topic of activism, public debate and research in the US context. In recent years, European countries have also seen a growing social and academic debate about the way racialized minorities are policed. Based on ethnographic research in Amsterdam, this article argues that in order to understand such racialized policing, we have to go beyond a narrow focus on the police itself, and instead examine the broader institutional landscape tasked with security. This institutional landscape is made up of penal and welfare actors who together enact what I call diffuse policing. Such diffuse policing envelops targeted persons and spaces in a dense web of surveillance, and disciplinary and reform interventions that are hard to escape or challenge. This article explores the cumulative effects of this dense security landscape, and argues that it produces significant inequalities among youths in Amsterdam.

## Introduction

‘Handled with care’ is the slogan of the Top600 Approach, the security policy that targets 600 of Amsterdam’s repeat offenders. The slogan captures the combination of coercion and care that characterizes many of the security policies that target troublesome and criminal youths in Amsterdam. This article examines the thick institutional ‘youth and security’ landscape that envelops such youths, and explores the diffuse forms of policing it inaugurates. I argue that the shared institutional focus on particular places and categories of people and behaviours produces a very uneven reach and impact of security policies. Even if these policies are mostly not violent and not merely repressive, they result in highly unequal experiences of the state among Amsterdam’s youths.

In the US context, racialized policing and police violence has been a longstanding topic of activism, public debate and research (see, e.g., Alexander, 2011; Bowling and Marks, 2015; Rios, 2011). Scholars have also started to document racialized policing in continental Europe (see, e.g., Body-Gendrot, 2010; Bonnet and Caillault, 2014; Çankaya, 2012; Fassin, 2013; Mutsaers, 2014; Peterson and Åkerström, 2014). In order to understand discriminatory forms of policing, most of these studies have focused on the police institution itself. In this article, I argue that the focus on the police institution is too narrow to understand the discriminatory effects of policing in the Netherlands. In dense welfare states such as the Dutch one, many more actors are involved in the surveillance, disciplining and reform of ‘risky’ subjects than the police. To understand the effects produced by such diffuse, networked policing, we need to examine the broader institutional landscape through which security policies are envisaged and practised. In this article, I use the case of Amsterdam’s Diamantbuurt, a neighbourhood notorious on account of its unruly young men with Moroccan-Dutch backgrounds, to explore the types of surveillance and discipline enacted by what I call ‘diffuse policing’, and examine the forms of inequality produced in the process.

Drawing on 18 months of qualitative research in the Diamantbuurt, this article provides a view of security policy from the bottom up, charting the everyday life of security policies and, importantly, the stacking of policies that effectively produces a thick ‘youth and security’ landscape. The research focused on the broad range of actors involved with the Diamantbuurt, including journalists, politicians, policy officials, street-level professionals and young and old residents from various backgrounds. Research was conducted from 2011 to 2012 with the assistance of Hakima Aouragh; we employed a range of qualitative methods and gathered multiple kinds of data, including interviews, media reports, policy documents, and fieldwork observations.

While informed by the entire range of data, this article mainly draws on two sets of interviews with key actors in the youth-and-security domain. The first set consists of 20 interviews with professionals involved with youth and security policies: policy officials and street level professionals, such as the community police officer and youth workers. The other set of interviews was with eight local young men with Moroccan-Dutch backgrounds, between their late teens and late twenties, who were part of the youth-and-security target group. This group is generally hard to reach and does not trust authorities. Most of these interviews were with two interviewees at the time and were made possible through the intervention of trusted social workers, who were also present during the interview. These two sets of interviews often spoke to each other directly, with professional interviewees discussing particular young men we had interviewed, and vice versa. In addition, we had many informal conversations with these same outreach social workers about these young men and the youth-and-security policy network, and participated in various events in the youth-and-security domain, from the regular open house at Streetcornerwork, the organization for outreach social work, to an anniversary event of that same organization attended by various youth-and-security partners (see De Koning, 2015) and a public debate about the Top600 Approach.

In the following I first discuss institutional legacies and changes in security policies in the Netherlands. I then introduce the site of this case study, the Diamantbuurt, and the reasons for its notoriety. After a sketch of the contours of the local security landscape, I explore how this institutional landscape attempts to monitor, discipline and reform particular groups of young men. I use interviews with some of the young men who are its prime targets to explore how this ‘youth and security’ landscape manifests itself in their lives. I end with a reflection on the effects of diffuse policing, and the inequalities it produces.

## Racialized policing

Ethnic profiling was long considered non-existent or insignificant in the Netherlands (Van der Leun and Van der Woude, 2011: 447–8). In recent years, however, supervisory bodies and academic observers have begun to discuss the prevalence of ethnic profiling (Eijkman, 2010; Mutsaers, 2014; see Amnesty, 2013, for an excellent overview). Anthropologist Sinan Çankaya’s study (2012) of proactive policing practices in the Amsterdam police force is the most detailed study to date. According to Çankaya, proactive policing, which aims to prevent offenses that may happen in the future, involves the routine categorization of the population into probable victims and probable perpetrators. Young Moroccan-Dutch men with a ‘street look’ and Eastern European men were prime targets of such proactive policing.

Çankaya documents not only a police culture replete with negative stereotypes of Moroccan-Dutch young men, but also points to a number of structural features of the police organization that stimulate ethnic profiling. For instance he discusses the ‘Show-off Approach’, which entailed a focus on non-white young men who drive big cars, thus creating an official incentive for a Dutch variety of ‘driving while black’. Çankaya also demonstrates the crucial role of the police information system. In the context of proactive policing, police officers note information they expect to be potentially useful. The initial choice to make a note on a specific person often relies on the racialized ideas about possible victims and perpetrators discussed above. When information on an individual is entered into the system, it will stimulate other officers to also note down details on this particular individual, resulting in a chain of notes on that person in the police information system, without any wrong doing or even marginally suspect behaviour on his part. The initial racialized judgement call defining someone as a potential person of interest continues to incite further police attention.

In his ethnographic study of a Parisian anti-crime squad, Didier Fassin (2013) argues that the policing of social housing projects has become a way to assert social order rather than maintain public order. He demonstrates that this policing is deeply racialized and discusses at length the entanglement of probability thinking with racial stereotypes that together steer police officers towards such racialized policing. He concludes:

It is the institution of law enforcement, and society more broadly, that produce the racialized categories that officers put into action on the ground, transforming minority youths into suspects, just as political discourse has done in recent decades in linking immigration and crime. (Fassin, 2013: 168)

Çankaya and Fassin’s studies elucidate the institutional setting that leads to a focus on particular racialized groups of youths, and the procedures and routines that further consolidate the focus on these groups. They also explore the adversarial type of interactions between racialized youths and police that are produced through such institutional settings and routines. Their work documents the prevalence of ethnic or racial profiling in police work in France and the Netherlands, and draws attention to the societal and institutional contexts that help explain its seemingly common-sense nature and wide-spread occurrence.

Building on these important contributions, I argue that the policing of risky subjects is done through a dense institutional landscape performs a diffuse kind of policing that marries welfare to discipline, care to coercion and prevention to repression (cf. Gressgård’s (2016) notion of ‘welfare policing’). In line with observations by Simon Hallsworth (2006) and Paul Mutsaers (2014), I argue that if we want to examine racialized policing, we should turn to this wider institutional landscape.

## Security policies in the Netherlands

The diffuse policing I describe here fits in with a long history in the Netherlands with interventions in the lives of poor, urban families intended to monitor, discipline and reform them (see, e.g., De Regt, 1984). While such paternalistic interventions lost some of their appeal in the 1970s, paternalism is considered an enduring characteristic of Dutch governmental institutions (Duyvendak, 1999). Schinkel and Van der Berg (2011: 1928) argue that far-reaching governmental interventions have regained popularity in recent decades, albeit only for specific categories of citizens: ‘Paternalism is very much publicly supported, yet for other people: the urban “vulnerable” poor’. Many local security policies display a combination of care and coercion that seems to have become the hallmark of that part of the current Dutch welfare state that deals with what it considers risky populations (see, e.g., Schinkel and Van den Berg, 2011; Schilder, 2009). These ‘risky populations’ are most often poor, often non-white, and are assumed to be found in big cities.

These recent forms of paternalism bring to mind Wacquant’s (2012) discussion of a bifurcated governmentality that is liberal at the top and paternalistic and punitive at the bottom. It also fits with Peck and Tickell’s analysis of ‘roll-out neoliberalism’ that entails ‘new modes of “social” and penal policymaking, concerned specifically with the aggressive reregulation, disciplining, and containment of those marginalized or dispossessed by the neoliberalization of the 1980s’ (2002: 389).

Since the 1990s such interventions have increasingly been framed in terms of security. The Dutch term *veiligheid*, which invokes both security and safety, has become increasingly important to the understanding of social problems and the formulation of a wide range of policies (Pakes, 2010; Schuilenburg and Van Swaaningen, 2013). Van Houdt and Schinkel (2014) describe how the shift from the more delimited notion of crime to the broader notion of security resulted in a drive to create more encompassing and comprehensive policies, in which security was to be the concern not only of various state institutions but also of civil society and citizens. This broad scope was expressed in the naming of such policies as the Integrated Safety/Security Policy, or Integraal Veiligheidsbeleid. The focus on the nebulous trope of security has been accompanied by a growth in the budget allocated for security-related policies; between 2002 and 2012 national expenditures on security went up from € 8.510 million to € 13.043 million.^1^ This Integrated Safety/Security Policy is implemented through a wide range of interconnected state and semi-state organizations.

The rise of this type of security discourse and related policies is intertwined with growing nationalist and anti-migrant sentiments and increasingly racialized understandings of social problems. In public imagination, and in less explicit ways in official discourses, *overlast* and crime have become firmly linked to what in the Netherlands are glossed as *allochtonen*, people with ‘non-Western’ migrant backgrounds, and to Moroccan-Dutch young men in particular (Pakes, 2004; Mutsaers, 2014).^2^ Ostensibly universal policies meant to tackle social problems are often specifically geared toward poor, non-white populations with migration backgrounds (Van Houdt and Schinkel, 2013).

As I demonstrate below, the thick institutional landscapes that pivot around the trope of security are central in the translation of racialized ideas about society and its problems – particularly associations between migration, minorities and crime – into actual policies and street-level practices. I use the term ‘racialization’ to capture such processes despite the fact that in the Dutch context ‘ethnicity’ is a more common term than ‘race’, in order to stress that the ascription of characteristics on the basis of group membership is a highly generalizing, involuntary and negative imposition. Different groups have been racialized at various historical junctures; in Amsterdam, at the time of research, Moroccan-Dutch young men were the key racialized other (De Koning, 2015).

## Dealing with the ‘notorious’ Diamantbuurt

For some three decades, public debates in the Netherlands have revolved around the alleged problems with people with migrant backgrounds, and the folly of earlier multicultural approaches (Prins, 2002; Van Reekum and Duyvendak, 2012). In 2004 Amsterdam’s Diamantbuurt became pivotal in these public debates after reporting in national daily *De Volkskrant* on a conflict between a couple – designated by the quintessentially Dutch pseudonyms Bert and Marja – and local ‘Moroccan’ youths who were hanging out just opposite from their house.^3^ It came to symbolize the allegedly beleaguered state of ordinary (white) Dutch in the big city, due particularly to the misbehaviour of young ‘Moroccan’ men (De Koning, 2013).

The Diamantbuurt area has a long history of boys and young men, most with Moroccan-Dutch backgrounds, hanging around in groups on certain corners and passageways in the neighbourhood. Some of these young men were involved in criminal activities. Police and policy circles first categorized their loose networks as a ‘nuisance-causing (*overlastgevende*) youth group’, and in 2009, they identified it as a criminal youth group, naming it the Van Woustraat Group after the name of the street where the youths congregated.

As I argue elsewhere, the Diamantbuurt became an icon of the alleged problems of the multicultural Netherlands, particularly related to ‘Moroccan problem youths’ (De Koning, 2013). As a consequence of its iconic status, the Diamantbuurt became a test case for local politicians, the district administration and the myriad institutions involved with the neighbourhood. Media attention put pressure on the administration and various institutions to intervene, since the trouble in the neighbourhood was taken as an indication of governmental failure, and its alleged inability or unwillingness to acknowledge and act upon problems with racialized ‘others’.

The conception of the Diamantbuurt as an exceptional problem neighbourhood instigated and facilitated an increased policy focus on the neighbourhood and prompted the formulation of spatially targeted policy initiatives, particularly within the so-called youth and security policy domain. The major challenge was how to deal with complaints about *overlast* – a term that refers to a wide range of behaviour, from hanging around, to littering, causing nuisance or harassing of passers-by, and is used most often in connection to youths (see Martineau, 2006: 102–4) – and the relatively high incidence of youth criminality in the area, both of which were understood as primarily related to Moroccan-Dutch male youths.

## The youth and security policy network

At the time of research, most policies were designed at the city level, while a district administration was responsible for the implementation of policy and the management of relations between (semi)government agencies and the population. Policies for specific neighbourhoods, in this case the Diamantbuurt, were developed at the district level; this involved close cooperation of various state and non-state actors (housing corporations, social work agencies) in the management of urban space, and entailed significant overlap between different policy domains, for example housing, social welfare and security.

In response to the media attention and public uproar caused by the Bert and Marja affair, the district commissioned research and policy advice and created extra capacity to oversee a new approach to problems with Diamantbuurt youths. Over the years, this evolved into a ‘youth and security’ (*jeugd en veiligheid*) policy that brought together a wide range of actors and agencies, and was overseen by officials from the city district’s youth and security division.

The policy was envisioned as a network linking many partners (*ketenpartners,* chain partners), from local authorities to youth work, outreach social work, debt relief, truancy officers, police and public prosecution, and housing corporations, all of which shared information and coordinated their activities under the auspices of the district authorities. Much of its attention was focused on the Diamantbuurt.

Similar youth and security policies have been developed in other cities in the Netherlands (see, e.g., Kaulingfreks, 2015). They have much in common with English multi-agency partnerships designed to tackle local problems of crime and disorder, described by Daniel McCarthy (2014). Such close cooperation has been hailed as a solution to the fragmentation of governmental approaches in various institutional domains, from security to immigration control (Mutsaers, 2014) and welfare.

This youth and security policy network developed an extensive range of measures directed at youth nuisance and youth criminality, drawn, in part, from programs and practices of many other social agencies. At the end of 2011, for example, an action plan targeting the Diamantbuurt’s ‘hard core’ of criminal young men included measures from the realms of security and policing, social work, and physical environment and housing.^4^ The policy’s target group varied from as many as 180 youths to as few as 20, depending on the specific aspect of youth and security spectrum that was emphasized (*overlast* or crime) and the scale that was prioritized (neighbourhood, district or city).

Besides close cooperation among various institutional actors, information gathering became another key governmental technique. Willem Schinkel (2011) describes the Dutch state’s escalation in ‘actuarial government’ through the establishment of databases and surveillance registers that enable intricate calculations of risk and are supposed to allow state actors to spot early signs of trouble in order to avert unwanted future outcomes. According to Schinkel (2011: 367), such archives

are part of the rhetoric of ‘prevention’ and ‘early detection of risks’. Yet at the same time, they facilitate the policing of families and criminalization of subjects with multiple archival registries.… [T]he current ‘archive fever’ is a form of *prepression* that combines prevention and repression.

The gathering and sharing of detailed information on young people within the network was indeed an important aspect of the district’s security approach. At the district level, various agencies, from ‘street coaches’ to social work and different branches of the police, as well as individual residents, contributed to the mapping of unruly youths and their behaviour in public space. At the time of research, the district authorities’ project coordinator for youth and security kept continuously updated street maps that recorded the movements and contacts of some 180 youths from the district who had been written up in connection to *overlast* in public space. Her team decided on appropriate trajectories for these unruly youngsters. This approach entailed forms of prepression, as causing nuisance in public space was taken as an early sign of future deviance, and acted upon accordingly; it included both repression and prevention strategies. A surveillance and archiving machinery was put into place to spot these early signs, and to act upon their occurrences to prevent undesirable futures.

In tandem with the security policies at the district level, the city administration developed the Top600 Approach, in place since 2011. With its emphasis on surveillance and data gathering, and preventative incursion into the lives of designated young male habitual offenders in order to preclude future deviant behaviour, the Top 600 Approach is a textbook example of prepression.

The Top600 focused on a list of 600 young men who were suspected of involvement in so-called high-impact crime (mostly street robberies, theft and house break-ins). They were not listed on account of ongoing investigations or the suspicion of concrete misdemeanours, but because they were considered repeat offenders who were likely to continue committing crimes. The Top600 combined the efforts of 30 organizations, including police, city and district administration, the judiciary, rehabilitation services, and public health and social work agencies. The Top600 team designed a tailor-made combination of care and coercion for each subject, i.e. per person included on the list, to be overseen by one ‘director’. Depending on the nature of the problems, the director would be recruited from the police, the probation office or the public health authorities. These directors would be able to organize help for their ‘client’, in terms of treatment, work and even housing, as long as the client stuck to the plan that was laid out for him.

The Approach was presented as an exemplary governmental technique with its close cooperation between a wide range of governmental organizations from social work and public health authorities to public prosecution and the police. The Top600 Approach was portrayed as a model to be followed in the streamlining and improvement of government in other domains.

Those included in the Top600 list were often described as ‘the most violent young criminals’ in news media*.* However, as far as I could ascertain, the list included youth who had committed only very light crimes as well as those involved in heavier cases. A lawyer who worked for a number of young men on the Top600 list argued that the criteria for inclusion were far from straightforward, an impression that was confirmed in various informal conversations with policy actors. An actual conviction was only one of many criteria, which also included number of ‘contacts’ with the police and arraignments over a number of years (see Groenendaal and Van der Venn, 2012). The lawyer said she saw a clear correspondence between the target group of the Top600 and those singled out in public discourses. ‘From what I have seen, the guys that I know who are on the list really fit the current political climate. They are all those types that people react to strongly, of whom they are afraid and feel they should be taken off the streets.’

The Top600 Approach was portrayed as the answer to the limitations of regular approaches, as ‘thinking outside the box’ to tackle the persistent deviancy of young men throughout the city. It presented a combination of repression and care that was meant to leave the selected habitual offenders no choice but to either cooperate fully and be guided out of their life of crime or to be constantly monitored, have their daily routines disturbed and be pestered by the police, a practice dubbed Very Irritating Policing. Being closely associated with Mayor Van der Laan, the Top600 was presented as the hard-nosed (Social Democratic) response to the purported left-wing neglect of the problems with multicultural society, and its alleged naivety and softness with regards to ‘*allochtonen’* (Prins, 2002).

Even though the Top600 was a citywide policy, it was fully integrated into the district’s security approach and relied on much of its institutional infrastructure. The policies initiated at the district and city levels included a number of the same policy officials and street-level professionals such as social workers and police officers, and had some of the same young men as their target. They, moreover, relied on the same information streams and often coordinated their actions.

## Blurred boundaries

At the district level, representatives from police, district and city authorities and youth and outreach social work agencies exchanged information and calibrated their approaches to what they considered the most at-risk and troublesome subjects in the area. Their policies combined care and coercion, prevention and repression. Their concerted efforts created a network that allowed for a diffuse policing shared among many agents, and led to a blurring of boundaries between social and welfare work and policing, between repressive agents of the state, and those that extend a helping hand and trusted ear.

Developments in the youth-and-security network illustrate this blurring of care and coercion. In the years following the 2004 Bert and Marja incident, the regular youth work open house that had provided the young men with a space to meet and work out was cancelled. These unruly young men were no longer to be served out of the regular youth budget. Instead, outreach social work organization Streetcornerwork started organizing an open house as a way to establish relationships with this hard-to-reach group. This open house was presented as a way to recruit clients and thereby facilitate the real work of helping solve the young men’s problems, whether they be debts, unemployment or homelessness. Instead of being able to resort to regular youth work, the local young men were thus targeted by outreach social work with the aim of rehabilitating them. It is telling that, despite their indispensable role in the youth-and-security network, Streetcornerworkers were regarded with suspicion by other network partners on account of their close relations with their clients.

In contrast with the tougher stance in the social work domain, the Top600 Approach introduced forms of soft policing akin to those described by Daniel McCarthy (2014), which added to the blurring of care and coercion. The Top600 Approach was meant to assist repeat offenders choose another life, among other things by providing help in finding housing, work or a traineeship. Top600 directors were to have regular meetings with their ‘customers’ to keep them on track and help them better their lives. However, as was the case in McCarthy’s study policing, such caring advice was always backed up by the threat of repression if advice was not followed and young men would stray from their prescribed path.

The diffuse policing enacted through this policy network targeted one particular group, Moroccan-Dutch young men who were at the heart of the Diamantbuurt’s notoriety. As I demonstrate below, the discriminatory effects of such policing were both dispersed and ubiquitous.

## Diffuse policing

Our interviews with Moroccan-Dutch young men from the Diamantbuurt give a sense of the interconnectedness and cumulative nature of the youth and security landscape discussed above. These young men shared a sense of injustice in connection to their treatment by the authorities, particularly the police. A local youth worker in his late 30s, Sayid, who grew up in the area and knows many of the families of the unruly young men who are his charges, said: ‘In my days, you were not monitored. Now you’re being followed by the district and the police’. A young local concurred: ‘You really become para [paranoid]’.^5^ His distrust extended not only to district officials and police officers, but also to local youth workers, whom many of these young men considered to be in cahoots with the police.

This exchange reflects the changes in local security policies that had occurred in the years leading up to the research. Especially after the 2010 ascendency of the right-wing VVD party in the district administration, security policies had been stepped up, transforming the neighbourhood into a hostile space for local young men, particularly those of Moroccan descent. CCTV-cameras had been installed, hanging around in groups began to be severely policed and any transgressive behaviour was swiftly and heavily sanctioned.

Below I draw on our interviews with some of these young men to explore their experiences with youth and security policies. These young men spoke almost exclusively about the youth and security landscape in terms of surveillance and repression. Even more ‘caring’ interventions, such as those of family visitors or Top600 directors, and welfare figures like the local youth workers, were understood as repressive. The Streetcornerworkers were the only institutional representatives whom this group of young men trusted.

### Street coaches

Rachid was born and raised in Amsterdam’s Diamantbuurt. When I interviewed this 18-year-old in 2012, he straddled the border between being a poster boy for local youth work and being seen too often in the company of the local drug dealer. After talking about the regular ID checks to which he is subjected in his neighbourhood, Rachid turned to another encounter with the authorities:

One time the District came to my door.

Anouk: *Really, why?*

[They said:] ‘We see Rachid in the company of older youths too often. He’s out in the streets too late at night’. I mean, that’s none of their business!… I thought it was all a load of crap! It happened to a guy next door, he had to be home [by 4 PM] for three months.…

Anouk: *Why?*

He was also someone who was seen with the wrong kind of guys, according to them, that is.

The encounter with the ‘District’ that Rachid describes concerns a family visitation conducted as part of the street coaches program. At the time of research, the district authorities contracted a foundation to run a street coaches program that combined surveillance, intervention and data gathering regarding youth nuisance in the streets with home visits to unruly youths. According to its website, this Foundation for Tackling Nuisance Amsterdam, or Stichting Aanpak Overlast Amsterdam (SAOS), was established in 2006 in order to ‘combat youth nuisance and antisocial behaviour in a decisive and efficient manner’. It claims to fill the gap between youth work and the police, and states that it focuses on groups of youths who display antisocial behaviour that in most cases does not constitute a legal offense but is rather a transgression of social norms.^6^ As one of the founders explained to me:

As street coaches we operate between on the one hand the police, and on the other youth work; we correct [the youths] when needed, but also advise and listen to them.… But it is clear we’re not their pals, we do not build a relationship of trust with them.

District authorities could buy SAOS’s services to patrol areas that were perceived as suffering from youth nuisance. These street coaches were selected from a pool of private security guards employed by a large security company, and then further employed by SAOS. Their task was, on the one hand, to prevent and intervene in youth nuisance in public space. On the other, they were expected to identify perpetrators and more generally keep tabs on the public behaviour and social lives of local youngsters. The latter information was noted down in daily reports, which were sent to local police stations and district policy officials. Street coaches were a major source of data for the street maps that were kept by the district authorities.

SAOS also employed so-called family visitors, like the one who visited Rachid, to follow up on those youths who have been identified twice in connection with public nuisance. Family visitors were meant to enter families into a three-month-long program intended to improve their child’s problem behaviour. A family program could include measures like the curfew that was imposed on Rachid’s neighbour.

Such ‘behind the front door’ programs have been critiqued as an assault on people’s privacy and civil rights (Schilder, 2009). The selectivity of such programs – targeting particular spaces, groups and behaviours judged as potentially troublesome – has received less attention (but see Schinkel and Van den Berg, 2011). In the South District, they were employed in those areas that were seen to suffer from youth *overlast*, particularly in the Diamantbuurt. While their work was not explicitly racialized, the people, behaviours and spaces that street coaches targeted both drew on and reproduced racialized notions of troublesome behaviour and risky populations.

### Everyday policing

Danny, in his 40s, with short blond hair and a friendly expression, had been the Diamantbuurt’s *buurtregisseur* or community police officer for a number of years at the time of research. In an interview in 2012, I asked him about the measures that were taken to combat *overlast.* To my surprise he said that at the moment there was no *samenscholingsverbod* or ban on gathering in specific places. Yet a number of the local young men I spoke with discussed such bans, and argued that they stayed away from the neighbourhood’s public spaces on account of the many fines they had received for violating that ban.

Danny explained: ‘The municipal bylaw [APV, Algemene Plaatselijke Verordening] does offer a number of tools to take repressive action on those spots where they hang out.… We use them if necessary. Aimless hanging around against a porch is one of them.’ He continued, describing how they try to keep the young men in the neighbourhood in check:

What we do is look very closely at those spots in the neighbourhood that deserve extra attention. If CCTV-cameras are installed at the Smaragdplein, we say to each other, ‘What’s going to happen next?’ Because you have a good sense of the neighbourhood, you can predict that it will probably shift to the Lutmastraat, where you have a small playground. And within two weeks that set [of youths] was there. What do you do then, as police? You go check it out, what is happening there, how do they behave? And, in fact, without doing anything concrete, [the set] moves again, simply [because the police are] passing by… because these guys themselves feel uncomfortable. And don’t think now that we pass by ten times an hour.

When we talked to young men who were targeted by these measures, there seemed to be quite some confusion concerning the actual measures that were in place. This vagueness did not diminish but rather added to the measure’s efficacy, creating a perpetual uncertainty regarding what was forbidden and what was allowed among the young men who knew themselves to be under police scrutiny. It also gave individual police officers more leverage in reprimanding or fining youths. Such ambivalence was, at least in some cases, intentional, rather than the result of a faltering communication. A social worker told me that the district authorities encouraged a repressive approach and stimulated police officers to use their discretionary power to act on small infringements. The Top600 even formally includes this kind of ‘Very Irritating Policing’ (their term) as a way to deal with Top600 youths who do not comply with the program.

A lawyer at a firm that defended several young men from the Diamantbuurt told me that one of her clients had been fined five times in one day for minor and rather common transgressions, such as riding his bike on the sidewalk. She says she feels that the treatment of these young men has changed radically: ‘Especially this harrying. It started already before the Top600. I remember a postcard the guys in the Diamantbuurt found in their mailbox, some eight years ago… It said, “We are watching you”. So I think the police have been on their case for quite a while.’ She saw a clear connection between the changes in security policy in Amsterdam and the public focus on particular types of nuisance and crime – rowdy, constant presence in public space and so-called high-impact crime – and on particular types of perpetrator: young men with non-white, minority backgrounds.

Amir and Mohamed (Mo), both in their early 20s, discussed their experiences with the changing security policies in the neighbourhood. Amir, the more talkative of the two, took the lead:

Amir: Those cameras don’t influence me, I can walk right by them 50 times.

Mo: You do feel they are looking over your shoulder, but you get used to it. There is also a ban on hanging out.

Amir: I did get fined a number of times, but I didn’t pay them.

Anouk: What were the fines for?

Amir: For assembly, for leaning against things; there are various fines. Boom, he prints it for you. You’re also not allowed to hang on the ledge of the Edelsteen [the local community centre]. Danny really likes to fine Mo.

Mo: But my lawyer makes them disappear again.

Amir: They drive by and give you a fine. When you come home, you find out that you have a fine. You feel so powerless. Of course, you could lodge a formal complaint or go to court, but well… This way they force you to get into trouble.

Amir said that he no longer walks through the neighbourhood with his head raised high, like he used to. ‘Much has changed’, he said. ‘It used to be a nice neighbourhood, much more lively. Guys no longer dare to hang in the streets’. Mo agreed: ‘Now they are at the shisha lounge or coffee shop [cafes where one can buy and smoke soft drugs] all day’.

According to most residents, everyday policing was stepped up significantly after the neighbourhood’s rise to notoriety in 2004. Everyone had stories to tell about the extra rounds made by the police car, or the policemen on bikes who would check out groups of youths hanging out in the neighbourhood. Peter, a well-known neighbourhood resident, liked to recount the time when he put some chairs on the central square during Ramadan and invited a number of the local young men to sit and chat. After a few hours of conversation, the young men remarked that they would never be allowed to sit like this in the square if it wasn’t for Peter’s presence.

This spatial intensification and targeted approach is complemented by other types of policing that, unlike the work of local police officers, is not specific to the area and not based on familiarity with its particular young people. As Çankaya has demonstrated, this type of policing easily lends itself to ethnic profiling. Take Mostafa’s story. Mostafa is exceptional among his friends from the neighbourhood on account of his successful educational career and clean rap sheet. In an interview in 2012, he told us that he, nonetheless, is often stopped by police officers:

When I ride my scooter, there are always officers on motorbike who want to see my licence plate and ID. [When I ask,] ‘Why?’ ‘Traffic control.’ ‘Am I the only one?’ ‘This time, yes.’ In the summer this happens to me like five times per week.… They check prior convictions, whether the scooter is stolen, unpaid fines.

Mostafa’s story is not an isolated incident. In the context of proactive policing, traffic laws are often used as a pretext to investigate the identity of persons that police officers consider of interest (Çankaya, 2012). Those judgment calls on the part of police officers largely rely on racialized categorizations of the population in likely perpetrators – young, male, with minority backgrounds – and potential victims: the body of ordinary citizens that needs to be protected from the latter.

### Top600

While the Top600 Approach was presented as a fight against high-impact crime and the city’s persistent young criminals, it also paid a good deal of attention to the much publicized *overlast* of young people by targeting many of the young men who were often out in public. The Top600 added a personalized focus to the more general criminalization of conduct in public space in the Netherlands (Martineau, 2006) and the policing of categories of likely perpetrators, since it directed attention to the conduct of specific young men who were included on the list.

Danny, the community police officer, explained that ‘the Top600 [list] gets undivided attention.… Everyone knows who’s on [the list], everything is registered. We have a complete picture, almost hour by hour.’ Mo, who was on the Top600 list, told us that he was followed and monitored wherever he goes. He said police officers regularly called out to him by name in public, outing him as a person of interest for the police: ‘When the police drive by and call you by name, you really feel put on the spot. [When they say] “Hey Mo!”, I say, “Hey asshole”.’

I asked Martin, a police officer assigned to the Top600, what he thought about Mo’s feeling of being harassed by the police. I mentioned the case of being called out by name in public. Martin noted that doing so can be a form of intervention, especially if someone on the list has made it clear that he does not want to cooperate. However, he also noted that officers in the regular police service generally do not know details about a person’s trajectory. They might simply act on the Top600 status, irrespective of whether the person in question ‘behaves’ (and should therefore be left alone) or refuses to cooperate (which would justify the ‘Very Irritating Policing’ approach). He described how officers might respond to a complaint about nuisance (*overlastmelding*) that brings them to youths who are part of the Van Woustraat Group:

They ask for their IDs, call into the precinct and hear that four of them are in the Top600. The uniformed police will then probably say, ‘Hey guys, you’re in the Top600. We don’t have anything on you now, but mind you, we’re keeping an eye on you’. So, yes, they do get that stamp among police officers, and I can imagine they don’t like it.

When I asked Martin whether the fines for small infringements were not disproportionate, he said:

I sometimes also run a red light, but I am not in the Top600. And if I was, I would do anything to take care that I got off that list. That may be quite harsh, but, apparently, it does work, because the nuisance in that area has stopped and many of the guys are doing quite well.

Intensified policing of the neighbourhood and of particular figures like the ‘members’ of the Van Woustraat Group or Top600 ‘clients’ likely raised the number of recorded offenses in the area. This is not only because the chances of criminal activities being discovered increased, but also due to the proliferation of acts that were seen to require police action and constitute an offense. Moreover, as Femke Kaulingfreks (2015) argues, such police presence may set off a vicious circle between close surveillance and repression and unruly, disturbing behaviour of these young men. Anecdotes from the Diamantbuurt indicate that intensified policing also contributed to a rise in incidents that were directly related to that police presence, such as charges for insulting a police officer or resisting arrest.

The law firm that represented a number of young men from the Diamantbuurt was highly critical of the Top600 Approach. One of the lawyers had started the long process of trying to get one client off that list, among other things with recourse to the European Charter for Human Rights. The lawyer I cited earlier felt the Approach became a ‘self-fulfilling prophecy’.

Because you’re on the list and you’re from that neighbourhood, it means you’ll be arrested quickly. And you’ll be sent home after two days because they actually have nothing on you, but you do have a citation next to your name. When that happens a few times, they’ll say, see, you have a lot of police contacts.… Even though you are actively sought out by the police because you are on that list. Because they have a description of the subject: guy on a black scooter with a black jacket, and, well, that can be anyone in Amsterdam. And then they start stopping guys from the list, that’s just how it works.

Like the other security policies discussed here, the Top600 Approach did not explicitly employ racialized definitions of the problem or of its target group, and the program officer I interviewed even vehemently denied that they took ethnic background into account. However, at the time of the interview, the young men from the Diamantbuurt constituted the largest single subgroup in the Top600, and the lawyer’s comments give us a sense of the subtle ways in which the policy, in responding to racialized understandings of social problems, reproduces a racialized focus on particular types, behaviours and places.

## Producing inequality

The youth and security programs and measures discussed above impact the way various categories of persons are monitored and disciplined and what interventions are designed to reform them. The range of organizations that was involved together created what I have called diffuse policing that combined intensive monitoring, care and repression in highly focused, targeted ways. The cumulative effects of the security landscape I have sketched above produce highly unequal experiences of the state.

As illustrated by the specific examples discussed above, particular areas that were perceived as problematic saw a thickening of the youth and security landscape, which made young people in those areas far more likely to be registered as risky or troublesome and become the target of interventions by a variety of state agents. In her discussion of a similarly deprived neighbourhood in Utrecht, Femke Kaulingfreks (2015) even speaks of a penal panopticon.

In the specific, limited areas in which street coaches were deployed, unruly behaviour was likely to have very different consequences than it would in areas that were seen as unproblematic and not requiring street coach surveillance. Unruly behaviour in ‘problematic areas’ could easily lead to the inclusion in the district’s register of problem youths and could result in the start of a family program, whereas such behaviour was unlikely to have consequences in ‘unproblematic’, wealthier areas in the same city district. Moreover, everyday policing practices in the Diamantbuurt had turned the neighbourhood into a hostile space for young men with Moroccan-Dutch backgrounds, who, more generally, were habitually categorized as potential perpetrators. They were fined for small transgressions, and the neighbourhood provided easy grounds for criminalization. Finally, the Top600 Approach had more personalized effects in terms of experiences with the state. It, however, also drew heavily from the same city spaces and categories seen as particularly problematic. When included on the list, young men would get full, often unwanted, invasive attention from actors across the youth and security landscape.

These unequal experiences with the state were produced by the spatial focus of youth and security policies, and their attention to specific types of persons and behaviours. In line with Simon Hallsworth’s argument, we could understand these policies as control regimes that, without being framed in racial terms, ‘either licence racial targeting, or which appear guaranteed to reproduce it’ (2014: 308). Moreover, the security policies and practices discussed here shared many of the routine categorizations of the populace as likely perpetrators and potential victims that, as Çankaya (2012) notes, are mobilized in proactive policing and which reproduce a racialized common sense about crime.

The diffuse policing enacted by the dense institutional youth and security landscape enveloped the targeted youths in a dense and sticky net of surveillance, discipline and interventions that was hard to escape or contest. This is well illustrated by the case that one of the young men, supported by the law firm mentioned earlier, brought against the city of Amsterdam in 2014 to fight his inclusion in the Top600 list. The case was brought to an administrative law court since, in a legal sense, the Top600 Approach was merely an administrative measure. The lawyer for the city argued that the only issue that could be contested was the exchange of information between the various organizations that cooperated in the context of the Top600 Approach. Otherwise, the plaintiffs should turn to specific agencies or institutions that they felt had overstepped their mandate or crossed lines of privacy or equity.

This line of thinking was repeated in a public meeting in 2016 intended to discuss the Top600 as it was going into its fifth year. A policy official with the Top600 extolled the efficacy of the Approach, stressing that, over time, it had been successful in bringing the radically different worlds of care and repression together. Forty organizations now worked together under its auspices, with very high success rates, for instance in terms of the significant decrease in the number of crimes committed by those on the list. And yet, the policy official argued, in a juridical sense, we only exchange information.

## Conclusion

European grassroots activists and critical social scientists increasingly join their US counterparts in addressing racialized forms of policing. This article has argued that to understand the effects of racialized policing in Europe, we need to look at the wider institutional landscape involved in monitoring, disciplining and intervening in the lives of ‘problematic’ or ‘at-risk’ populations, and pay attention to the diffuse policing it enacts.

Using the case of the Diamantbuurt, this article demonstrates that the Dutch welfare state, with its extensive apparatus and legacy of benign paternalism, is key to how security policies translate the racialization of social problems common in public discourses into actual policies and urban everyday life. The security programs discussed here all combined forms of care and coercion, manifesting the new paternalism observed in the Netherlands more generally. They relied heavily on information gathering and detailed surveillance in order to signal risks and try to avert future calamities. Such prepression programs were enacted by a wide range of governmental actors, from public health authorities to social work, rehabilitation services and the police and prosecutors, whose concerted actions together produced a dense network of information gathering, surveillance and intervention. Policy networks that brought together welfare and disciplinary actors enacted what I have labelled diffuse policing. Drawing on my ethnographic findings, I have argued that, as a result, particular categories in particular places, and oftentimes even particular individuals, are intensely monitored, and if the policy network so decides, treated to an insistent combination of care and coercion.

While these policies did not explicitly target racialized groups, they were often designed with particular groups or problems in mind. In Amsterdam, in the context of virulent debates about the ‘failure of multicultural society’ and the ‘trouble with *allochtonen*’, security policies were designed to showcase a serious engagement with the type of people, incidents and places identified with such issues. They were, moreover, implemented in a spatially specific manner, targeting particular ‘problem’ areas. These foci were shared by youth and security policies at various levels that complemented and reinforced one another. Together they resulted in an institutional landscape that intensively monitored and insistently intervened in the lives of particularly Moroccan-Dutch boys and young men from the Diamantbuurt and environs.

The accretion of such policies, which in themselves were often well intended, had far-reaching effects for those who were its main targets. These enveloping, invasive institutional interventions were hard to escape, contest or challenge, since they were designed to finally act decisively on the people and issues that various governmental actors had been unable to solve and that represented a highly politicized test case for government. We may read this as the Dutch equivalent to the policing of the projects described by Fassin (2013), which had as its ultimate goal to reassert the social order. To combat the era’s perceived number-one problem – unruly, street-oriented Moroccan-Dutch young men who may to varying degrees be involved in criminal activities – much seemed allowed, including stretching juridical boundaries and state powers.

## Notes

1. Source: CBS. See: http://statline.cbs.nl/Statweb/publication/?DM=SLNL&PA=80162ned&D1=0-29,40,46&D2=a&VW=T

2. *Allochtoon* (pl. *allochtonen*) is originally a bureaucratic term that distinguishes those with at least one foreign-born parent from an autochtoon, a native Dutch person. There is a further differentiation between non-Western and Western allochtonen based on country of birth (see Yanow and Van Haar, 2012). In everyday usage, *allochtoon* is almost always used to refer in an imprecise fashion to those people who used to be labelled ‘foreigners’ (Geschiere, 2009).

3. ‘Moroccan’, ‘Turk’, ‘Surinamese’, etc., are ethnic labels commonly used in the Dutch context, alongside the umbrella terms *allochtoon* and *autochtoon*. In their vernacular use, they are understood to refer to locals, often Dutch citizens, with particular ethnic backgrounds. When using ‘Moroccan’ I use inverted commas to highlight that these are vernacular terms. In my own analysis, I rely on the term Moroccan-Dutch to indicate how people would be ethnically identified.

4. Response to written questions by city council member Ornstein on 9 May 2011 regarding violent criminals in the Diamantbuurt (Beantwoording schriftelijke vragen van het raadslid mevrouw S.H.M. Ornstein van 9 mei 2011 inzake gewelddadige criminelen in de Diamantbuurt). Amsterdam, 20 July 2011; see: www.amsterdam.nl/publish/pages/341121/687_11.pdf

5. I have used pseudonyms for most persons cited in this article.

6. See the mission statement on the SAOS website: http://www.aanpakoverlast.nl (accessed 3 January 2017).
